# Natural Killer Cells: Potential Biomarkers and Therapeutic Target in Autoimmune Diseases?

**DOI:** 10.3389/fimmu.2021.616853

**Published:** 2021-02-19

**Authors:** Elena Gianchecchi, Domenico V. Delfino, Alessandra Fierabracci

**Affiliations:** ^1^VisMederi srl, Siena, Italy; ^2^Infectivology and Clinical Trials Research Area, Primary Immunodeficiencies Research Unit, Bambino Gesù Children's Hospital, Istituto di Ricovero e Cura a Carattere Scientifico, Rome, Italy; ^3^Section of Pharmacology, Department of Medicine, University of Perugia, Perugia, Italy

**Keywords:** immune homeostasis, self-tolerance, immunoregulation, autoimmunity, natural killer cells

## Abstract

Autoimmune diseases recognize a multifactorial pathogenesis, although the exact mechanism responsible for their onset remains to be fully elucidated. Over the past few years, the role of natural killer (NK) cells in shaping immune responses has been highlighted even though their involvement is profoundly linked to the subpopulation involved and to the site where such interaction takes place. The aberrant number and functionality of NK cells have been reported in several different autoimmune disorders. In the present review, we report the most recent findings regarding the involvement of NK cells in both systemic and organ-specific autoimmune diseases, including type 1 diabetes (T1D), primary biliary cholangitis (PBC), systemic sclerosis, systemic lupus erythematosus (SLE), primary Sjögren syndrome, rheumatoid arthritis, and multiple sclerosis. In T1D, innate inflammation induces NK cell activation, disrupting the Treg function. In addition, certain genetic variants identified as risk factors for T1D influenced the activation of NK cells promoting their cytotoxic activity. The role of NK cells has also been demonstrated in the pathogenesis of PBC mediating direct or indirect biliary epithelial cell destruction. NK cell frequency and number were enhanced in both the peripheral blood and the liver of patients and associated with increased NK cell cytotoxic activity and perforin expression levels. NK cells were also involved in the perpetuation of disease through autoreactive CD4 T cell activation in the presence of antigen-presenting cells. In systemic sclerosis (SSc), in addition to phenotypic abnormalities, patients presented a reduction in CD56^hi^ NK-cells. Moreover, NK cells presented a deficient killing activity. The influence of the activating and inhibitory killer cell immunoglobulin-like receptors (KIRs) has been investigated in SSc and SLE susceptibility. Furthermore, autoantibodies to KIRs have been identified in different systemic autoimmune conditions. Because of its role in modulating the immune-mediated pathology, NK subpopulation could represent a potential marker for disease activity and target for therapeutic intervention.

## Introduction

Natural killer (NK) cells are a heterogeneous population of innate lymphoid cells (ILCs). Different from B and T lymphocytes, ILCs do not express the type of diversified antigen receptors and are principally tissue-resident cells representing ~5–15% of the circulating lymphocytes in human (1). They are able to release cytokines and are characterized by natural cytotoxicity. NK cells have been identified in the early 1970s and, as suggested by their name, are able to exert spontaneous selective cytotoxic activity toward cells under stress, including virus-infected cells and cancer cells. Different from cytotoxic T lymphocytes, to conduct the anti-tumor function, NK cells do not need prior antigen exposure or previous specific immunization (2). For a long time, these cells were considered short-lived effector cells (3); however, they also show characteristics of the adaptive immune system, including the expansion of pathogen-specific cells, the generation of long-lasting “memory” cells that can persist upon cognate antigen encounter, and the ability to induce an increased secondary recall response to rechallenge (1). The initially recognized function of NK cells was their ability to kill cancer cells of hematopoietic origin (1). In particular, they exert elevated cytotoxicity toward tumoral cells and virus-infected cells through a critical balance of signals transmitted by a wide repertoire of activating and inhibitory receptors defined as killer cell immunoglobulin(Ig)-like receptors (KIRs) (Table 1). NK cells are classified into different subsets on the basis of their function or the nature of their ligands. Hence, signals from multiple receptors have to be integrated to sense the environment and respond properly. The majority of activating and inhibitory receptors share conserved sequences in their cytoplasmic portions, an immunoreceptor tyrosine-based activation motif (ITAM) and an immunoreceptor tyrosine-based inhibitory signaling motif (ITIM), to transmit signals (4). Activating NK cell receptors, including NK group protein 2 family member D (NKG2D); natural cytotoxicity receptors (NKp30, NKp46, and NKp44); DNAX accessory molecule-1 (DNAM-1); and the co-receptors NTB-A, 2B4, NKp80, and CD59 (5), are critically involved in NK cell activities. They recognize stress-induced ligands expressed on cancer cells and allow NK cells to kill them by releasing cytotoxic granules containing perforin and granzyme B or engaging death receptors tumor necrosis factor (TNF)–related apoptosis-inducing ligand or Fas ligand, or through antibody-dependent cellular cytotoxicity (6). In order to promote a productive response, a critical threshold of activating signaling that exceeds the counterbalancing influence of the inhibitory receptors must be achieved. As opposed to T lymphocytes and other adaptive immune cells, NK cells do not need prior sensitization for their cytotoxic activity. With respect to inhibitory receptors, those involved in human leukocyte antigen (HLA) binding are the most investigated. The engagement of ITIM by the ligand avoids cytotoxicity through the transmission of inhibitory signals. Main inhibitory receptors include the CD94/NKG2A receptor, the KIR family in humans (4), and the Ly49 family in mice (7). Remarkably, most of the mature NK cells are characterized by the presence of at least one inhibitory receptor (KIR or NKG2A) for self HLA class I antigens; however, a limited number of NK cells not presenting inhibitory receptors are anergic. The process of NK cell maturation, defined as NK cell “licensing” or “education,” modulates the repertoire of inhibitory receptors present on NK cells (8). Inhibitory KIRs and NKG2A are not only involved in limiting the cytotoxicity of NK cells, but they also play a critical role in NK cell education. More specifically, inhibitory signals halting the activation of NK cells and preventing cytotoxicity in case of ligand encounter are usually due to the constitutive binding of KIRs and HLA class I molecules present on autologous normal cells, avoiding the killing of autologous healthy cells (9). Such interaction involves the extracellular domains and the peptide residues of the HLA class I molecule; the amino acid positions are responsible for its affinity (10) and the continuous interactions influence the NK cell potency. Only NK cells expressing receptors recognizing self HLA class I molecules acquire full functional potential, whereas the rest are either deleted or anergic.

**Table 1:** Killer cell immunoglobulin-like receptors (KIRs) and their cellular ligands.

**Inhibitory KIRs**	**Cellular ligands**
KIR2DL1	HLA-C C2
KIR2DL2	HLA-C C1, HLA-B*46:01, HLA-B*73:01, HLA-C C2
KIR2DL3	HLA-C C1, HLA-B*46:01, HLA-B*73:01, HLA-C C2
KIR2DL4	HLA-G
KIR2DL5	?
KIR3DL1	HLA-B Bw4, HLA-A*23,HLA-A*24,HLA-A*32
KIR3DL2	HLA-A*03, HLA-A*11, HLA-F
KIR3DL3	?
**Activating KIRs**	**Cellular ligands**
KIR2DS1	HLA-C C2
KIR2DS2	HLA-C C1, HLA-A*11:01
KIR2DS3	?
KIR2DS4	HLA-C*02:02, HLA-C*04:01, HLA-C*05:01, HLA-C*01:02, HLA-C*14:02, HLA-C*16:01, HLA-A*11, HLA-F
KIR2DS5	HLA-C C2
KIR3DS1	HLA-F, HLA-B*51

**Figure 1:**
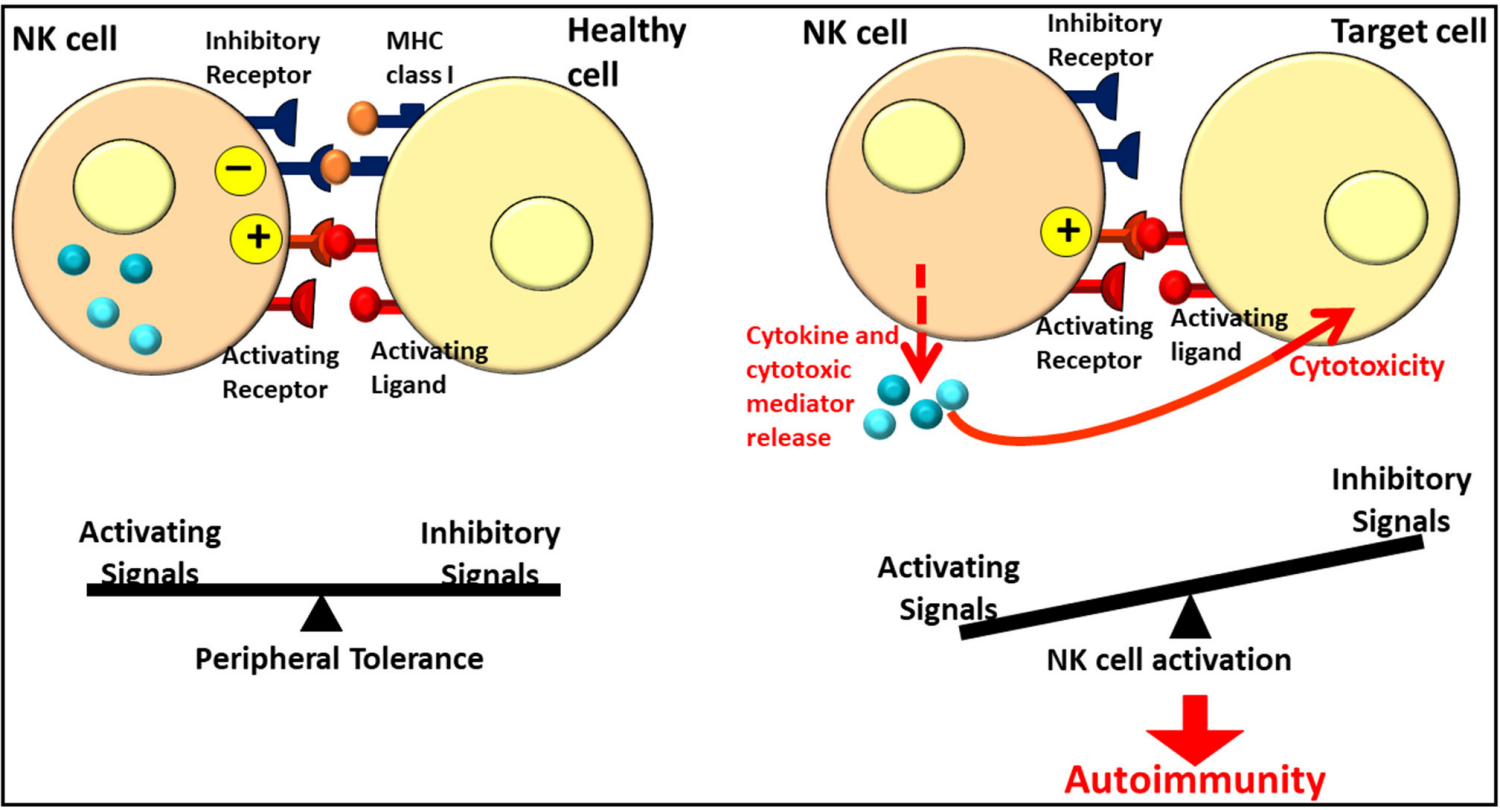
Role of NK cells in homeostasis and autoimmunity. Figure adapted from Ref. (12). NK, Natural Killer.

In addition to their anti-tumor spontaneous cytotoxicity, NK cells represent an interface between innate and adaptive immunity through the secretion of cytokines and chemokines, allowing the activation of local immune cells and the recruitment of additional immunotypes (11). Moreover, the role of NK cells in the control of the cellular immune responses promoting or downregulating them (12) and in homeostasis maintenance through the balance between the inhibitory and activating signals involving the activating and inhibitory KIRs has been recently recognized (Figure 1) (13). Granulocyte-macrophage colony-stimulating factor (GM-CSF), interleukin (IL)-13, and IL-10 (14–16) are the main immunoregulatory cytokines that are released. In addition, NK cells secrete chemokines such as chemokine (C-X-C motif) ligand 8 (CXCL8) (or IL-8), C-C motif chemokine ligand 2 (CCL2) [or monocyte chemoattractant protein (MCP)-1], CCL3 [or macrophage inflammatory protein (MIP)-1α], CCL4 (MIP-1β), CCL5 (RANTES), and CXCL10 [interferon (IFN)-inducible protein (IP)-10] (14, 17). Natural killer cells are phenotypically characterized by the expression of a surface marker CD56 while lacking CD3, but they do not represent a homogeneous population. More specifically, on the basis of their maturation status and functional characteristics, they can be distinguished into subpopulations (9). Depending on the CD56 relative expression, human NK cells can be distinguished into CD56^bright^ and CD56^dim^ subsets. CD56^bright^ and CD56^dim^ NK cells represent subsequent stages in the NK cell development. CD56^bright^ NK cells are generally considered to be the immediate precursors of CD56^dim^ NK cells in a linear-differentiation model (18) even though, for a long time, the two subpopulations were considered two separate lineages with different hematopoietic origin and properties (19). Once NK cell precursors leave the bone marrow, they move through the peripheral blood and join the lymph nodes (LNs), where they differentiate into CD56^bright^ NK cells under the influence of cytokines produced by stromal cells and DCs. Although CD56^dim^ NK cells are the predominant subset in the peripheral blood, CD56^bright^ NK cells are mainly found in the secondary lymphoid tissue and other tissues (20). Most of the NK cells (about 90%) are in fact CD56^dim^ and express elevated levels of FcγRIIIA (or CD16); in addition, they have significantly higher cytotoxicity and present much more perforin, granzyme, and cytolytic granules than CD56^bright^ cells (21). Only a minority (about 10%) of NK cells are CD56^bright^ and CD16^dim/neg^, with weak cytotoxicity before activation but constitute the most efficient cytokine producers endowed with immunoregulatory properties (20). Because of this ability, CD56^bright^ NK cells are identified as regulatory cells. In fact, cytokine release can modulate innate and adaptive immune responses. The principal cytokines produced in CD56^bright^ NK cells include IFN-γ, TNF-a, granulocyte–macrophage colony-stimulating factor, IL-10, and IL-13 (22). In addition, the cytotoxic ability following activation allows NK cells to be suppressive, as regulatory T (Treg) lymphocytes, toward autologous activated CD4^+^ T lymphocytes. The cytotoxicity effect is partially inhibited *via* the HLA-E expression on the target cells (23). Moreover, as reported by Morandi et al. (24), CD56^bright^ CD16^−^ NK cells secrete the immunosuppressive molecule adenosine (ADO) through a CD38-mediated pathway, a molecule implicated in the regulation of the immune response both in physiological and pathological conditions interacting with four different G protein-coupled receptors (A1, A2a, A2b, and A3). Since ADO receptors are expressed not only by NK cells but also by T and B cells, NK cells act as regulatory cells inhibiting autologous CD4+ T cell proliferation, similar to Treg cells. Such immunoregulatory function could be attenuated in the presence of autoimmunity or inflammatory states, as suggested by differences in ADO kinetics synthesis and in ADO receptor expression in the peripheral blood with respect to synovial fluid NK cells (24). The maintenance of homeostasis is critical to avoid excessive inflammation or the development of autoimmune responses. Even though the pathogenesis of autoimmune disorders is mainly due to T and B lymphocytes, NK cells have been recognized to be involved in the promotion and/or maintenance of altered adaptive immune responses or in peripheral tolerance mechanisms and, for such reasons, could be therapeutically exploitable in the context of T cell-mediated autoimmune diseases (1).

## The Role of NK Cells in Autoimmune Diseases

**Figure 2:**
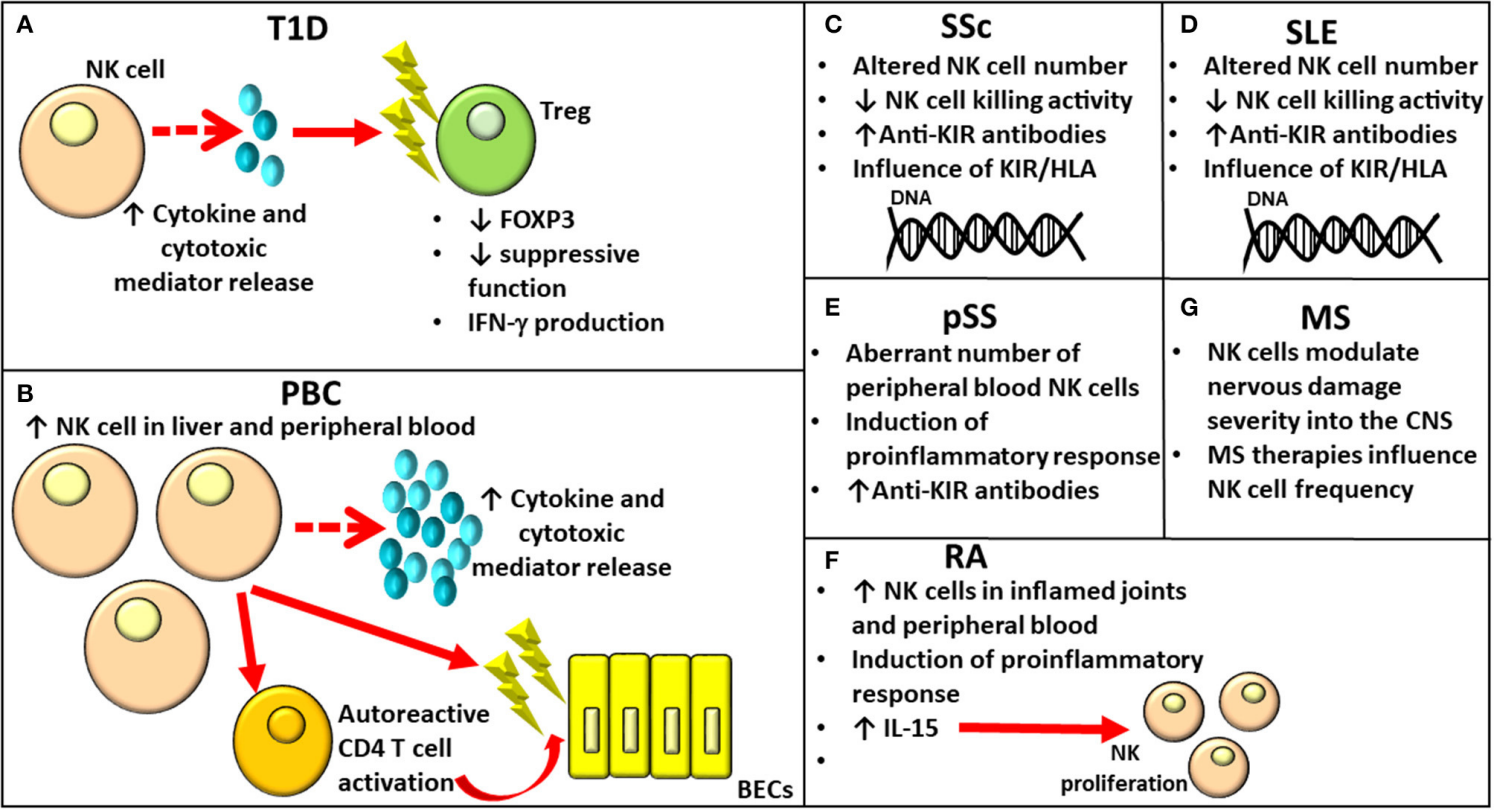
Role of NK cells in various autoimmune diseases. NK, Natural Killer.

Autoimmunity incidence has been increasing worldwide over the past 50 years. Autoimmune disorders have a multifactorial pathogenesis, involving both genetic and environmental factors. Although some autoimmune conditions have common pathogenic mechanisms, the exact mechanisms responsible for their onset remain to be elucidated. Their development is, however, caused by the failure of specific self-tolerance causing immune responses toward self-antigens (25). Over the past few years, the role of NK cells in shaping immune responses has been highlighted, reporting altered phenotype and aberrant cytotoxic capacity (Figure 2), even though their involvement is profoundly linked to the subpopulation involved and to the site where such interaction takes place.

## Type 1 Diabetes

Type 1 diabetes (T1D) is an autoimmune condition characterized by insulin-producing β cell destruction involving both innate and adaptive immune cells affecting glucose metabolism. β-cell death occurs for direct perforin/granzyme-mediated toxicity by CD8^+^ T cells and for the release of proinflammatory cytokines, such as IFN-γ, TNF-α, and IL-1β (26). As observed by MacKay (27) in diabetic Bio-Breeding/Worcester (BB/W) rats, the presence of pancreatic insulitis in this animal model allowed to hypothesize a cell-mediated immune pathogenesis for diabetes; the hypothesis was strongly supported by data obtained from studies based on immunological manipulation of BB rats. NK cells, whose function was enhanced in BB/W diabetic and diabetes-prone (DP) rats, were recognized to exert a cytotoxic function toward islet cells responsible for β-cell destruction and diabetes (Figure 2A). The role of NK cells in the onset of T1D is supported by findings from animal and human studies. The kinetics of different immune cells involved in the early phases of T1D development has been investigated in different organs (thymus, pancreatic-draining lymph nodes, and spleen) in the multiple low-dose streptozotocin (MLDSTZ) mouse model of T1D. The study has revealed that the first immune response involving DCs and B cells was followed by the involvement of T helper 1 (Th1) and cytotoxic T (Tc) cells. To elaborate further, from day 10, MLDSTZ mice presented a reduction in NK cells associated with a higher IFN-γ^+^ NK cell proportion (28).

The study conducted by Kallionpää et al. (29) focused on the identification of novel biomarkers that are able to predict the onset of the autoimmune response before the development of autoantibodies or resemble progressive β cell destruction in children. These authors found upregulated transcripts, including IL-32, before T1D-associated autoantibody positivity. Activated T lymphocytes and NK cells were mainly responsible for IL-32 upregulation as demonstrated by a single-cell RNA sequencing analysis.

The phenotypic analysis comparing immune subpopulations in human LNs and blood from patients with new-onset T1D revealed a high number of CD8^+^ naïve, FOXP3^+^ Treg, class-switched B cells, CD56^bright^ NK cells, plasmacytoid DCs, and CD4^+^ T cells in LNs (30). The presence of CD56^bright^ NK cells in human LNs was consistent with previous data (31). Conventional NK cells were virtually absent from LNs; however, many leukocyte subpopulations implicated in T1D onset showed different frequencies in the blood (30).

The importance of the NK cell role in the onset of T1D has also been recently demonstrated by Dean et al. (32). They showed that innate inflammation characterizing T1D induces NK cell activation disrupting Treg function. More specifically, certain genetic variants identified as risk factors for T1D influenced the activation of NK cells promoting their cytotoxic activity. In addition, activated NK cells showed a higher expression of NKG2A, NKG2D, CD226, T-cell Ig and ITIM domain (TIGIT), and cluster of differentiation (CD) 25. Furthermore, in case of co-culture with Tregs, the latter were characterized by downregulation of forkhead box P3 (FOXP3), production of IFN-γ, and loss of suppressive function (32) (Figure 2A).

Although NK functionality has been extensively analyzed, only a few studies have investigated the phenotype of cell subsets in patients with T1D (33). The group of Oras et al. (34) recently found a diminishment in the frequencies of CD16^+^CD56^dim^ and CD16^−^CD56^dim^ NK cell subsets in children with newly diagnosed T1D with respect to healthy controls. Barcenilla et al. (35) reported increased frequencies of two CD8^+^CD16^+^CD56^dim^ NK cell subsets in multiple autoantibody-positive children, who later progressed to T1D, compared to autoantibody-negative subjects. The same group conducted phenotypical analysis of peripheral blood mononuclear cell (PBMCs) from patients with T1D through the use of mass cytometry that revealed alterations in immune cell subtypes (36). In particular, a reduced frequency of CXCR3^+^T-bet^+^IFN-γ^+^ TEMRA cells within the CD8^+^ T cell lineage in the peripheral blood of newly diagnosed patients with T1D as compared to healthy controls was identified, in agreement with the observation of their infiltration into the insulin-positive islets wherein they constitute the most prevalent cell type responsible for pancreatic beta cell death (37). In addition, an expansion of activated mature/effector CD8^−^CD11c^+^CD16^+^CD56^dim^CD69^+^HLA-DR^−^ NK cells expressing IFN-γ was identified in newly diagnosed adults with T1D. Since long-standing subjects with T1D did not show an altered frequency of activated NK cells with a proinflammatory phenotype, such abnormality which is unique in newly diagnosed subjects with T1D could underlie the presence of an ongoing inflammatory condition (36).

## Primary Biliary Cholangitis

Primary biliary cholangitis (PBC) is an autoimmune liver disease mainly affecting women and characterized by the production of anti-mitochondrial antibodies (AMAs), lymphocytic infiltration of the portal tracts by several different immune cell subpopulations, and the gradual destruction of intrahepatic small bile duct epithelial cells (38). Although it mainly affects the liver, it is considered a systemic disease due to its association with several other conditions, such as Sjogren syndrome, scleroderma, thyroid disease, and autoimmune diabetes (39). Although CD4 T lymphocytes, through the secretion of cytokines, drive the activation of other immune subsets, such as autoreactive effector CD8 T and B lymphocytes, activated DCs, NK cells, NKT cells, monocytes, and macrophages initiate the autoimmune process in PBC pathogenesis. The activation of these subsets is responsible for the inflammatory state affecting the liver that can lead even to its failure (39). The role of NK cells in PBC pathogenesis mediating direct or indirect biliary epithelial cell (BEC) destruction has been supported by several studies (40). NK cell frequency and number were enhanced in both the peripheral blood and the liver of patients with PBC, and they were associated with increased NK cell cytotoxic activity and perforin expression levels (41) (Figure 2B). Autologous BECs would be killed by NK cells activated by IFN-α and toll-like receptor (TLR)-4 (42). Moreover, NK cells are involved in the perpetuation of a disease through autoreactive CD4 T-cell activation in the presence of Antigen-presenting cell (APC)s (43) (Figure 2B). Since the number of intrahepatic lymphocytes producing IFN-γ has been observed in patients with PBC, Ravichandran et al. (44) investigated their functional role in a murine model of sclerosing cholangitis reporting that CD8^+^ T cells and NK cells are the principal sources of IFN-γ. Furthermore, NK cell loss diminished CD8^+^ T cell cytotoxicity and liver fibrosis. According to the hypothesis that IFN-γ could impact the hepatic CD8^+^ T and NK cell phenotype and has a role in PBC pathogenesis, the complete absence of IFN-γ in Mdr2^−/−^x Ifng^−/−^ mice resulted in a reduction in NK cell and CD8^+^ T cell frequencies expressing the cytotoxic effector molecules of granzyme B and TNF-related apoptosis-inducing ligand (TRAIL) attenuating liver fibrosis. The recent study conducted by Hydes (45) reported that patients with PBC had circulating NK cells constitutively activated, enhanced levels of CD49a, and the liver-homing marker CXCR6 when compared to subjects with non-autoimmune chronic liver disease and healthy controls. RNAseq analysis conducted on NK cells from subjects with PBC revealed an upregulation of genes involved in IL-12/STAT4 signaling and metabolic reprogramming (45). The integrated genome-wide association studies (GWAS) and mRNA microarray analysis conducted recently by Ueno (46) have allowed to identify altered expression of genes related and not related to cell populations of the immune system, including NK cells, in subjects with PBC when compared to healthy controls.

## Systemic Sclerosis

Systemic sclerosis (SSc) is a connective tissue autoimmune disease characterized by inflammation, cytokine release, vascular alterations and damage, aberrant angiogenesis, and fibrosis. Although its etiology remains unknown, infectious, genetic (principally HLA class I and II alleles), and environmental factors (including organic solvents, industrial emissions, and asbestos), as well as epigenetic changes, play a role in the susceptibility and onset of SSc. Changes both in the immune and vascular systems are responsible for fibrosis affecting the skin and multiple internal organs. Fibrosis represents the principal cause for the elevated morbidity and mortality in SSc (47). Both B lymphocytes and T cells would be involved in the fibrotic process even though contradictory data regarding B and T cell number anomalies with respect to healthy controls have been reported (48–51). In addition to B and T lymphocytes, NK cell alterations in number and function were implicated in SSc pathogenesis (Figure 2C). The reduced NK cell number in the peripheral blood has been hypothesized to be due to the infiltration of NK cells in the affected tissues; on the contrary, investigations report an increase in circulating NK cell number in patients with SSc (52). Such discrepancies could be related to the disease stage and different clinical complications [Reviewed (Rev.) in (53)]. As reported recently by Gumkowska-Sroka through cytometric characterization of the principal immunocompetent cells in 46 adult subjects with SSc (54), patients affected with SSc presented a reduced NK absolute count with respect to healthy controls due to a lower NK cell frequency within the lymphocyte population. Concerning the putative relation between immune cell profile and disease, a significant reduction in NK cell percentage was observed in patients with SSc presenting with arthralgia with respect to those without. In addition, an investigation on the influence of the autoantibody profile [anti-Scl-70 and anti-centromere antibody (ACA)] on immune cell components revealed the presence of an increased NK cell number in patients who are ACA positive. The study of Van der Kroef (55) reported a reduction in CD56^hi^ NK cells in subjects with SSc compared to healthy controls in agreement with the phenotypic abnormalities previously observed by Almeida et al. (52). NK cells from SSc showed deficient killing activity (56). The investigation regarding the presence of anti-KIR antibodies conducted on 48 patients with SSc revealed their presence in 12.5% of patients with SSc as against 3% of healthy controls (57).

The influence of the activating and inhibitory *KIR/HLA* gene profile in SSc susceptibility has been demonstrated by studies conducted on different populations (Figure 2C). To elaborate further, a positive correlation with SSc was reported for *KIR2DS2*+*/2DL2-* in Germany (58); *KIR2DS1*+*/2DS2–* and *2DS1*+*/HLA-C2*+ in Canada (59); *2DS3*+, *2DS2*+*HLA/-C1*+, and *2DL2*+*/HLA-C1*+ in Turkey (60); *3DL1*+*/HLA-Bw4*^*Thr*^*-* in Iran (61); and *KIR2DL2* and *KIR2DS4del* in Mexico (53). However, a negative association with SSc was observed for *2DL3*+ in Turkey (60) and *2DL2*+ in Brazil (62). Although contrasting data regarding the association of KIR2DL2 with SSc were reported in two different populations, this gene correlated with susceptibility to autoimmunity in the Mexican population was also demonstrated for RA risk and response to treatment (62, 63). Machado-Sulbaran et al. (53) observed differences in the *KIR* and *KIR/HLA* frequencies in patients with SSc in the Mexican population with respect to other ethnicities. This finding could be related to genetic admixture and adaptation processes influenced by infectious agents and to environmental factors in different regions and continents (64). Even though the group of Machado-Sulbaran (53) observed a higher frequency of KIR*2DL2* correlated with most of the clinical SSc manifestations than other KIRs, this difference did not reach statistical significance. This study has, however, the limitation of the restricted number of enrolled patients. *KIR2DL2* might alter NK cell antifibrotic function, with the release of cytotoxic substances promoting cell lysis and cytokine secretion, including IFN-γ, responsible for the inhibition of liver fibrosis (56). With regard to *KIR2DS4*, which can codify for the full (*2DS4full*) and shorten (*2DS4del*) proteins, it is possible that alterations affecting protein structures can influence the activating role of the receptor (65) as demonstrated by the fact that SSc correlated with *2DS4del*, but negatively correlated with *2DS4full* [Rev. in (53)]. Additional studies on a large number of patients are necessary to investigate the putative correlation between *KIR2DL2* and the clinical disease characteristics.

## Systemic Lupus Erythematosus

Systemic lupus erythematosus (SLE) is a chronic multi-system autoimmune disease. Even though the etiology of SLE is still unclear, both genetic and environmental factors have been implicated in the disease mechanisms. Infections or environmental factors have been hypothesized to cause cell damage, promoting the exposure of self-antigens to the immune system and leading to B and T cell activation. Both innate and adaptive immune systems play a role in the pathogenesis of SLE. Several alterations in the NK cell number and in the surface expression of certain KIRs, as well as in the combinations of KIRs and HLA class I-ligands, have been identified in patients affected by SLE [Rev. in (57)] (Figure 2D). Several differences affecting both the expression and functions of the broadly expressed inhibitory receptor leukocyte-associated Ig-like receptor (LAIR)-1 have been observed in patients with SLE. To elaborate further, LAIR-1 reduced expression was observed in DCs and B lymphocytes; when the latter was stimulated with collagen, it showed a considerable reduction in Ig production. It has been hypothesized that such alterations could affect the immunoregulatory functions of LAIR-1, leading to the breakdown of immune balance and onset of SLE (66, 67). With respect to the genetic contribution on the development of SLE, *KIR2DL5* exerted a protective role for the onset of SLE and was associated with a higher risk of overall infections in patients with SLE than controls (68). In agreement with the previous data reporting an aberrant NK cell education process due to the presence of functional anti-CD94/NKG2A and anti-CD94/NKG2C antibodies in 3.4% of patients with SLE (69, 70), the frequency of autoantibodies to eight different inhibitory receptors involved in the modulation of NK cytotoxicity was investigated (57). A higher frequency of anti-KIR antibodies was reported in a group of 48 patients with SLE than in controls, accounting for 23% of patients compared to 3% in healthy subjects. Diminished degranulation and cytotoxicity of NK cells toward K562 tumor cells due to IgG from patients with anti-KIR-positive SLE were also observed. The reduced cytotoxicity of NK subpopulation could explain its weak activity observed in patients with SLE (71, 72) (Figure 2D). Furthermore, anti-KIR-autoantibodies reacting with >3 KIRs correlated with a higher disease activity, nephritis, increased IFN-α levels, and the presence of circulating anti-Sm and anti-RNP autoantibodies (57). In SLE, not all NK anomalies observed were related to autoantibodies or genetic variants, as reported by Suárez-Fueyo et al. (73) and Nehar-Belaid et al. (74). While the former noted that a downregulation of CD3ζ in NK cells shifted the cells toward a proinflammatory phenotype rather than affecting their cytotoxic function (73), the latter described a unique transcriptomic signature of principal cell types, including NK cells, absent in healthy subjects (74).

## Primary Sjögren Syndrome

Primary Sjögren syndrome (pSS) is a slowly progressing autoimmune condition wherein the exocrine glands are infiltrated by lymphocytes, principally B and T cells, causing a considerable loss of secretory function with consequent oral or eye dryness (75). In addition, patients with pSS present serum autoantibodies and hyperglobulinemia (76). However, limited data on the role of innate immunity in pSS are currently available. Even though a direct link between NK cells and the pathogenesis of pSS has not been identified in animal models with pSS so far, NK cells could have a modulatory activity in exocrine gland tissues and in the peripheral blood. This is demonstrated by the promotion of the inflammatory process observed in the salivary glands due to IFN-γ secretion caused by the interaction between epithelial cells and NK cells expressing NKp30 (77) (Figure 2E). Since contrasting data regarding changes affecting the proportion as well as the clinical relevance of CD56^high^ cells in patients with pSS are currently available (77, 78), further analysis has been conducted by Ming et al. (79), who hypothesized a correlation among a shifted balance affecting CD56 NK cell subsets and the immune status of subjects with pSS. Patients with pSS had a diminished frequency and absolute number of peripheral blood NK cells than controls (Figure 2E). In addition, there was not only an increase in CD56^bright^ NK to CD56^dim^ NK ratio but also such ratio correlated with serum IgG levels. However, it was negatively associated with complement C3 and C4 levels and not negatively associated with EULAR Sjögren's syndrome disease activity index (ESSDAI) in patients with pSS. Investigations on the ratio of CD56^bright^ NK to CD56^dim^ NK in other autoimmune diseases, such as SLE and IgG4-related disease (IgG4-RD), were conducted, reporting only a slight increase of CD56^bright^ NK subset, but no differences were observed in patients with IgG4-RD. These data support the possible use of the ratio of blood CD56^bright^ NK to CD56^dim^ NK as the diagnostic value specific for pSS in autoimmune conditions (79).

Namkoong et al. observed that serum autoantibodies present in patients with pSS bound muscarinic acetylcholine type 3 receptors (M3R) and positively correlated with leukopenia *in vitro* (80). To elaborate further, the viability of Jurkat T cells was not influenced by IgG collected from patients with pSS; conversely, it was consistently affected when primary NK cells were present. NK cells were considered to be involved in cell death responsible for leukopenia frequently observed in patients with pSS. Cell death would be the result of downregulation of plasma membrane-resident M3R and MHC class I molecules in leukocytes due to anti-M3R autoantibodies. In agreement with previous data on the identification of autoantibodies to KIR in different systemic autoimmune conditions (81), the investigation on a possible association between eight different anti-KIR antibodies and pSS, conducted on 119 patients with pSS revealed that autoantibodies to at least one KIR were present in 13 patients (10.9%) (Figure 2E). Furthermore, a significant higher frequency of anti-KIR-positive sera in patients with pSS with respect to controls was reported, and reactivity to each of the eight KIRs was observed in sera from patients with pSS (57). Even though the limited number of patients who are anti-KIR-positive could not allow to ascertain a possible correlation between the presence of KIR autoantibodies and disease activity, only patients with pSS with >3 KIR autoantibodies showed interstitial nephritis. Conversely, such clinical manifestation was present in only 1 of 106 patients with anti-KIR-negative pSS. So far, no data regarding the role of anti-KIR autoantibodies in nephritis onset are available; however, a correlation between patients with end-stage renal disease and a defect in NK cell education were reported by Prakash et al. (82). This led to the hypothesis that the renal disease onset of both autoimmune and non-autoimmune etiology could be related to both a genetic and an antibody-mediated interference with the education of NK cells.

## Rheumatoid Arthritis

Rheumatoid arthritis (RA) represents an autoimmune chronic inflammatory joint disease causing the inflammation of synovial membrane, cartilage, and bone destruction with consequent disability (83). Its molecular triggers have not been completely clarified yet. The observation of NK cell accumulation in inflamed joints with RA has supported the pathogenic role played by this subset through releasing proinflammatory cytokines, including TNF-α and IFN-γ, and interacting with immune and non-immune cells in the joints (Figure 2F). More specifically, they promote the co-stimulation of T and B lymphocytes and cytokine release by fibroblast-like synoviocytes; furthermore, in case of co-culture with monocytes, NK cells triggered osteoclastogenesis and bone destruction in arthritis (84). Nevertheless, contrasting opinions on the role of NK cells in the onset of RA are still reported (84, 85). Since they represent the principal producers of IFN-γ, they could play a protective/regulatory function through the lysis of activated macrophages and T cells, the blockage of the differentiation of osteoclasts, and thus the destruction of the bone. In addition, they are able to halt the differentiation of Th17, representing the principal T lymphocyte subpopulation in RA [Rev. in (86)]. Although multiple immunotypes play a role in the disease etiopathogenesis, the integrative analysis of GWAS and the expression of quantitative trait loci datasets recently conducted by Ping (87) depicted 25 NK immune-related pathways having a role in the onset of RA. The study of Schwanek et al. (88) investigated NK and T-cell subpopulations in case of different anti-rheumatic therapies. The study evaluated 508 patients affected with RA and revealed that, if rituximab, abatacept, and tocilizumab exerted no effect on lymphocyte subdifferentiation, TNF inhibitors and age instead meaningfully influenced the numbers of NK cells, as well as of T cells, T-helper cells, T-NK cells, and γδT cells. In light of these data, the use of age- and treatment-adjusted standard values for lymphocyte subpopulations during clinical trials and therapy of RA was envisaged.

In enrolled patients, the presence of *KIR2DL2* was associated with a risk to SSc; in contrast, this gene correlates with a lower risk of development of SSc in patients from Brazil. However, in western Mexico, *2DL2* has also been associated with the risk for RA and response to methotrexate treatment; this could suggest that in the Mexican population, *2DL2* gene is associated with susceptibility to autoimmunity (63). The response to methotrexate was also influenced by the expression of the *2DS4full* gene (89).

Lin et al. (90) aimed to investigate the phenotype and function of NK cells in patients with RA; their study reported not only higher NK cell percentages in the peripheral blood of patients than healthy controls but also increased serum levels of IL-15, a proinflammatory cytokine involved in NK cell proliferation and differentiation (Figure 2F). Consistent with previous observations in rats (91), the higher serum levels of IL-15 in patients with RA than in controls could play a pathogenic role in the maintenance of the inflammatory reaction characterizing RA by inducing IL-17, a proinflammatory cytokine. No differences in the percentage of resting NK cells associated with a consistent reduction of peripheral activated NK cells in patients with RA with respect to healthy individuals, were found by Elemam et al. (86). These results were in agreement with previous data reporting a defective activity of NK cells from patients with RA (92, 93) and in contrast with the observations conducted on the phenotype and the activity of NK cells in the synovial fluids (SFs) of patients with erosive deformative RA (DRA) and non-deformative RA (NDRA) (94). To elaborate further, in the SFs of subjects with DRA, a specific wide subset of activated synovial fluid NK (sfNK) cells was observed. In addition, patients with DRA and NDRA presented no differences in sfNK cell phenotypes, but DRA sfNK cells were characterized by a higher IFN-γ and TNF-α production upon IL-2 and IL-15 stimulation. These findings envisage the potential use of sfNK cells as a marker for disease severity of RA, and the identification of an elevated number of cells belonging to this subset could guide the clinician toward an earlier and more aggressive monitoring and pharmacological treatment. After controversial data regarding NK cell role in the development of RA were reported [Rev. in (86)], RA-specific genetic transcriptional signature in peripheral NK cells was recently investigated to select biomarkers potentially useful for the early diagnosis of this autoimmune condition and patient stratification. The differential expression of several NK receptors observed in subjects with RA by Lin and colleagues (90) could be used as promising biomarkers for the diagnosis of RA. To elaborate further, among the activating receptors, subjects with RA showed a reduced NKp46 expression on NK cells compared to healthy volunteers, and its expression on NK cells obtained from both patients with RA and healthy controls was modulated by IL-15; no differences in CD69 expression were revealed among the two groups, but defective response to exogenous IL-15 was observed in the former. Increased expression of the two inhibitory NK receptors CD158b and CD158e were characterized with NK cells from patients with RA. Exogenous IL-15 upregulated CD158b expression on NK cells from both patients with RA and healthy volunteers and CD158e expression only in the healthy volunteer group (90). Aberrant expression of LAIR-1 was noted on different cell populations, including osteoclasts and CD4+ T cells, and may be responsible for a poor inhibitory effect on T-cell activation, leading to a condition of uncontrolled inflammation and promoting the progression of autoimmune diseases. It has been supposed that LAIR-1 might behave as an anti-inflammatory compound in RA (67).

In addition, disease severity of RA was associated with the NK cell receptor expression. More specifically, NK cells from patients with RA affected by bone deformity and erosion showed diminished NKp46, perforin, and granzyme B expression compared to patients without bone erosion and deformity. This led to hypothesize that peripheral blood perforin and granzyme B expressing highly cytotoxic NK cells could migrate to inflamed joints in patients with RA presenting a more severe clinical picture. Elemam et al. (86) found differentially expressed genes in NK cells of patients with RA, with the overexpression of IL-1β, CXCL16, BTK, ITGB7, PECAM-1, CD56, and TLR10 and the downregulation of the p65 subunit of NF-κB RELA, IBTK, CCL2, and CCR4 genes with respect to controls. The expansion of CD56^bright^ NK cells was also previously observed in the peripheral blood of IFN-γ treated multiple sclerosis (MS) (95) as well as in patients with active and inactive SLE (95, 96). No differences were instead observed in CKLF, CXCL10, CXCR1, CXCR2, CXCR6, IL12RB2, IFNG, TLR3, and ICAM-1 expression between NK cells of patients with RA and healthy controls. *KIR* gene polymorphisms can be encountered among the genetic elements affecting RA susceptibility although contradictory results were reported in other studies [Rev. in (97)]. The meta-analysis conducted by Aghaei on 11 case-controlled studies involving various populations of Europe, Asia, and American nations (97) revealed a reduction in the risk of RA considerably correlated with *2DL3* due to its inhibitory role on the secretion of cytokines, such as IFN-γ causing the diminishment of T lymphocyte autoreactivity (98, 99). In addition, an association was seen also with *2DL5, 2DS5*, and *3DL3* genes, supporting their potential protective role. *KIR2DS5* represents an activating receptor; its protective role identified in RA and in other autoimmune conditions might be related to linkage disequilibrium (LD). Likewise, the expression of NK CD16^+^56^+^ receptor molecules have been analyzed to identify reliable clinical response biomarkers in patients with RA receiving rituximab therapy to control the effects of the treatment. Such analyses have allowed to find a limited number of CD16^+^ in subjects with RA, a biomarker representing a reduced lower cytotoxic activity of NK cells (100).

## Multiple Sclerosis (MS)

Multiple sclerosis is the most common immune-mediated pathology, affecting the central nervous system (CNS) characterized by non-homogeneous immunopathological and clinical phenotypes. In many cases, it causes irreversible disability. The factors responsible for such differences have not been clarified yet; however, genetic factors may play the lead role. NK cells migrating into the CNS are able to modulate nervous damage severity (Figure 2G). However, these cells not only promote the adaptive immune responses but also are even involved in the prevention, ending, and/or limiting them. NK cell modulation is strictly linked to the subpopulation involved, the site, and milieu wherein such interaction occurs [Rev. in (101)]. As for other autoimmune conditions, MS research aimed to identify potential biomarkers of different treatments before the onset of clinical/radiological signs of MS activity. CD56^bright^ NK cells have been, therefore, identified. To elaborate further, an increase in their number has been observed following therapy with IFN-γ, alemtuzumab, dimethyl fumarate, after autologous hematopoietic stem cell transplantation, and it is even more elevated in patients responding to fingolimod (Figure 2G). Such increase could be correlated with a stronger regulatory activity in some cases. No reduction in CD56^bright^ NK cell number characterized untreated subjects with MS; however, the observation that several treatments induced a rise in the absolute or relative number of CD56^bright^ NK cells in the peripheral blood sustains that this subpopulation occupies a critical position in the modulation of adaptive immune responses. It is plausible that therapies for MS could impact CD56^bright^ NK cell frequency by enhancing cytokine levels in case of reduced CD4+ and/or CD8+ T-cell numbers or inducing cytokine release by targeting cells/direct stimulus on NK cells. Such finding is, however, affected by limitations due to the loss of a cutoff value for CD56^bright^ NK cell number (102). A particular easy test in a blood sample depicting a reliable marker of treatment efficacy would enable promp modification of the therapy, redirecting to choose other therapeutical strategies in case of no changes in the immune subset identified. In addition, the long-term evaluation of such biomarkers could be relevant in assessing the risk of future disease activity. In case of relapsing-remitting MS, Gilmore et al. (103) have characterized long-term repopulation of peripheral immune cells after alemtuzumab treatment. Changes were detected in several different lymphocyte subpopulations with an increase of CD4+ T cells, B cells, and NK cells, whose surface phenotypes were specific of regulatory subsets and Tregs with higher regulatory capacity. It is plausible that the mechanism of action of alemtuzumab responsible for its clinical efficacy could be due to the complex network of interactions that involves regulatory populations in each compartment. In addition, NK^bright^ cell changes induced by alemtuzumab treatment might be responsible for the durable effect of the therapy as demonstrated by their persistence for at least 2 years after treatment (104).

## NK Cells in Other Autoimmune Conditions

NK cell-mediated responses have been involved not only in the pathogenesis of the previously described autoimmune diseases but also in other autoimmune disorders, including autoimmune hepatitis (AIH) (105), Behçet's disease (BD) (106), inflammatory bowel disease (IBD) (107), vitiligo (108), uveitis (109), and myasthenia gravis (110). Even though they have been recognized to play a role in the development of these disorders potentially altering the balance of immunity through the modulation of cytokine secretion or affecting the interaction with other cells, for most of these conditions, the regulatory mechanisms that modulate NK cell function have not been clarified yet (109).

## NK Cells as Potential Therapeutic Targets

An increasing number of studies indicate the important role played by NK cells in the pathogenesis of different autoimmune conditions, and for such reasons, different molecules targeting NK cell function regulation have been identified as potential therapeutic targets. Among the molecules which could represent potential targets for obtaining beneficial effects in the treatment of autoimmune diseases, there are the soluble CD83 (sCD83), a molecule able to negatively regulate NK cellular function in experimental autoimmune uveitis (EAU) (109) and the chemokine-receptor-3 (CXCR3) isoform B whose inhibition prevented melanocyte apoptosis and the further activation of T lymphocytes (108). This finding would allow not only the prevention but also the treatment of vitiligo during the initial stages of melanocyte destruction. One potential limit in the study is represented by the fact that CXCR3B can be investigated only in human samples since such isoform is not present in rodents not enabling the study of neither its expression nor its role in animal models with vitiligo (109). On the basis of the observations that patients with SLE are often characterized by anti-KIR autoantibodies that could be involved in the decreased NK cell cytotoxicity constituting a risk factor for the development of lupus nephritis, it is possible that the use of anti-KIR autoantibodies could be of clinical relevance. Regardless, additional investigations are necessary to fully elucidate the link between NK cell education and nephritis to clarify if both a genetic and an antibody-mediated interference with NK cell education could induce the onset of renal disease of both autoimmune and non-autoimmune etiology (57).

An increasing number of studies supported the role of T-cell immunoglobulin and ITIM domain (TIGIT), an inhibitory receptor expressed principally on activated T cells and NK cells as a checkpoint inhibitor of the immune system and its use as potential novel therapeutic target (111). TIGIT pathway involving T-cell responses has been implicated in the regulation of SLE (112, 113). This is supported by the findings about a significant reduction in TIGIT-expressing NK cells in patients with SLE with respect to controls, which were negatively correlated with disease activity and that TIGIT^+^ NK cells presented a considerably reduced functional potential compared with TIGIT^−^ NK cells. In addition, the blockage of the TIGIT pathway using functional anti-TIGIT monoclonal antibody reestablished IFN-γ secretion of NK cells (114). These data, in addition to the observation from animal models with RA (115) and that the TIGIT expression levels on human NK cells was associated with functional heterogeneity among healthy individuals leading to different susceptibilities to infection, autoimmune disease, and cancer, support the importance of TIGIT as a powerful negative regulator of NK cells in SLE, whose activation could represent a potential therapeutic strategy (116). NK cells can represent potential therapeutic targets as also demonstrated by their response to low-dose IL-2 immunotherapy (117). GM-CSF-secreting NK cells have an important role in the inflammatory cellular cascade involved in the amplification of joint inflammation and in the persistence of autoantibody-driven arthritis (118). GM-CSF is known to modulate pathogenic inflammation in autoimmune disorders, including RA and MS. Different therapeutic approaches can be adopted for the treatment of autoimmune conditions, including NK function modulation by targeting their activating and/or inhibitory receptors, IL-18 inhibition, or anti-GM-CSF therapies in case of the treatment of RA (118). NK cells can be also indirectly modulated by the administration of anti-IL-18R antibody as demonstrated by the reduction of infiltrating NK cells in the inflamed eyes and spleens of mice affected by EAU and the relief of the symptoms of EAU (119). The compound apremilast, which is able to inhibit the enzyme Phosphodiesterase 4 (PDE4) and halt the synthesis of several cytokines, including TNF-α and INF-α *in vitro*, has been investigated in the phase III trial (120), resulting in an effective treatment of active psoriatic arthritis (PsA) with ameliorations in enthesitis and dactylitis for up to 3 years. Among the treatment for ankylosing spondylitis (AS) currently available, there are several TNF-α inhibitors, targeting the proinflammatory cytokine TNF-α, released by a variety of cell types including NK cells. TNF-α inhibitors are characterized by different molecular composition and immunological effector functions (121).

Further elucidation of the aberrant functions of NK cells in the different autoimmune diseases will contribute to finding new therapeutic targets for these disorders.

## Concluding Remarks

It is evident that our ability to modulate NK cells for therapeutic purposes might depend on a deeper knowledge of the biology of these cells and their link with autoimmunity.

Recent investigations, aimed to identify immune components that could be used as potential biomarkers of disease activity of different autoimmune conditions, have focused on NK^bright^ cells. Changes in the NK cell number and in the other subset of lymphocytes expressing CD38 have been associated with the prevention or an improvement of pathological processes in the animal models with collagen-induced arthritis (CIA) (122). The immunophenotyping of cells from whole blood using flow cytometry can constitute an important tool to monitor immune blood cells and changes in the NK cell subset as potential novel biomarkers to be used also in the follow-up of patients with autoimmune disorders (103). Even though contrasting results regarding the protective and promoting role of NK cells for the onset of autoimmunity are available so far, further investigations are necessary to obtain a better understanding of their role and the precise cellular and molecular mechanisms which are involved for their possible use as targets to drive immune responses and prevent autoimmune processes or, if the autoimmune response is already present, therapeutic interventions.

## Author Contributions

EG wrote the manuscript. AF contributed to writing and supervised the content. DD critically revised the manuscript. All authors contributed to the article and approved the submitted version.

## Conflict of Interest

EG was employed by VisMederi srl. The remaining authors declare that the research was conducted in the absence of any commercial or financial relationships that could be construed as a potential conflict of interest.
